# Association of composite dietary antioxidant index with cardiovascular disease in adults: results from 2011 to 2020 NHANES

**DOI:** 10.3389/fcvm.2024.1379871

**Published:** 2024-06-28

**Authors:** Run Wang, Weijun Tao, Xiaobing Cheng

**Affiliations:** Department of Cardiology, The Third People’s Hospital of Hefei, Hefei, Anhui, China

**Keywords:** oxidative stress, composite dietary antioxidant index, cardiovascular disease, NHANES, cross-sectional studies

## Abstract

**Background:**

Oxidative stress is a known pathogenic mechanism in cardiovascular disease (CVD), yet the association between dietary antioxidants and CVD in the general population remains underexplored. This study leverages data from the National Health and Nutrition Examination Survey (NHANES) to investigate the association of a composite dietary antioxidant index with CVD in US adults.

**Methods:**

Analyzing data from 25,997 adults (2011–2020 NHANES), we employed weighted generalized linear models, subgroup analysis, threshold effect analyses, and sensitivity analysis to assess the association between dietary antioxidants and CVD. Nonlinear associations were explored through a restricted cubic spline, with gender-specific stratification and threshold effect analysis to identify critical inflection points.

**Results:**

Increasing levels of the composite dietary antioxidant index corresponded with decreased CVD prevalence (*P* < 0.001). In all models, weighted generalized linear models revealed a consistent negative association between CVD prevalence. And in Model 3, Quartile 4 had a 29% lower CVD prevalence than Quartile 1[0.71 (0.59, 0.85), *P* < 0.001]. Meanwhile, the findings of the unweighted logistic regression model demonstrated stability. Various characteristics such as sex, age, race, PIR, education, BMI, alcohol consumption, hypertension, hyperlipidemia, and diabetes did not influence this inverse association (*P* for interaction >0.05). Notably a nonlinear association was observed, with a significant inflection point at 3.05 among women.

**Conclusion:**

This study demonstrates a strong negative association between the composite dietary antioxidant index and CVD prevalence, suggesting the potential protective role of dietary antioxidants. These findings underscore the need for prospective studies to further understand the impact of oxidative stress on cardiovascular health.

## 1 Introduction

Cardiovascular disease (CVD) stands as the predominant cause of mortality among adults globally, accounting for approximately one-third of all deaths each year (1). The impact of CVD on public health is profound, directing significant attention towards understanding how to reduce its morbidity and mortality (2). Consequently, there is an urgent need to delve deeper into identifying additional risk factors and preventive measures for CVD.

Central to the progression of CVD is oxidative stress (OS), which impairs endothelial cell function through increased production of reactive oxygen species (ROS) (3). Nitric oxide (NO) interacts with these reactive oxygen species (ROS), which lowers the bioavailability of NO. This reduction in NO levels affects the vasodilatory response, which is essential for preserving vascular homeostasis and degrades endothelial function (4). Concurrently, inflammation synergizes with OS, exacerbating atherosclerosis progression (5). Emerging research highlights oxidative stress as a key player in the pathogenesis and progression of diseases such as pulmonary arterial hypertension (PAH), atherosclerosis (AS), and myocardial infarction (MI) (6–8). Despite this, the clinical application of antioxidant drugs in CVD prevention and treatment remains limited, shifting focus to the potential of antioxidant nutrients obtained from daily diets.

The Composite Dietary Antioxidant Index (CDAI) is established as a scientifically valid and reliable instrument for evaluating the overall antioxidant capacity in daily diets (9–11). This index intricately amalgamates a multifaceted score derived from six pivotal antioxidant nutrients, encompassing vitamins A, E, C, zinc, selenium, and carotenoids (12, 13). The CDAI highlights its importance in the assessment of nutrition. Recent studies underscore the significance of the CDAI. Investigations have revealed CDAI's role in reducing lung cancer risk in male smokers (14), protecting against osteoporosis in individuals aged 40–85 years (15), and mitigating depression development in U.S. adults (16). A study by Yang found that individuals with type 2 diabetes mellitus who consumed a diet rich in antioxidants experienced notably lower mortality rates from cardiovascular diseases (17). Additionally, a negative association between CDAI and various cardiovascular conditions, including hypertension, coronary heart disease, and heart failure, has been observed (18–20). However, we have found no studies that directly explore the association between the CDAI and the overall prevalence of cardiovascular diseases in the general population. To address this gap, our study utilizes data from the National Health and Nutrition Examination Survey (NHANES), aiming to enrich our understanding of antioxidant nutrients in CVD prevention and management.

The primary objective of our study is to investigate the potential association between CDAI and cardiovascular disease in U.S. adults. This exploration aims to contribute valuable insights into future strategies for the prevention and treatment of CVD.

## 2 Methods

### 2.1 Study population

The National Health and Nutrition Examination Survey (NHANES), a pivotal initiative of the National Center for Health Statistics (NCHS), plays a crucial role in evaluating the health and nutritional status of the United States' adult and child populations. This comprehensive program combines detailed interviews with thorough physical examinations to gather its data. Ethical compliance is a cornerstone of the NHANES methodology; the NCHS Research Ethics Review Board has rigorously approved all research protocols. Additionally, we ensured strict adherence to ethical standards by meticulously securing written informed consent from every individual. For transparency and public engagement, the NHANES has made its exhaustive research methodologies and datasets publicly accessible at www.cdc.gov/nchs/nhanes/, facilitating further research and analysis.

In the study, we analyzed data from 45,462 participants from the NHANES database covering the years 2011 to 2020. To focus the research on adults, individuals under 20 years of age, totaling 19,182, were omitted. Furthermore, the analysis excluded 236 participants due to incomplete questionnaire data, which was essential for diagnosing cardiovascular diseases. Additionally, 47 participants with missing information on education and smoking were excluded. Consequently, the final sample size for the study comprised 25,997 participants.

**Figure 1:**
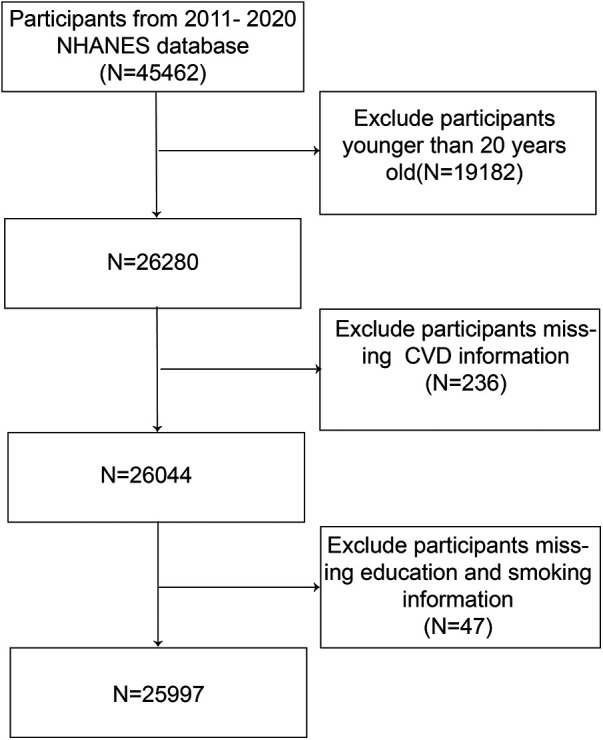
The process flow diagram for the systematic selection method.

Figure 1 illustrates the process of participant selection for the study through a detailed flow diagram.

### 2.2 Definition of the composite dietary antioxidant index and cardiovascular disease

In this analysis, the derivation of CDAI data was executed via a 24-h dietary recall interview. This method involved detailed documentation of all foods and beverages consumed by the participants in the 24 h preceding the interview, facilitating an accurate estimation of the nutrients. However, they do not include nutrients obtained from dietary supplement intakes, antacids, or medications. We used a modified version of the CDAI formula in our investigation to precisely measure the total amount of dietary antioxidant consumption (14, 21, 22). The six essential antioxidant vitamins included in this improved CDAI formula are carotenoids, zinc, selenium, vitamin A, vitamin C, and vitamin E. The formula for calculating the CDAI is as follows:

$$\overset{6}{\underset{i=1}{\sum }}\frac{\mathrm{Xi}-\mathrm{ui}}{\mathrm{Si}}$$

The antioxidant intake was denoted by Xi, a mean Xi of the entire cohort was represented by ui, and the Si showed the standard deviation (SD).

In this study, the classification of CVD was based on participant responses to the survey questionnaire. A diagnosis of CVD was considered if participants reported any of the following conditions: angina, myocardial infarction (MI), stroke, congestive heart failure (CHF), or coronary heart disease (CHD). Consequently, any affirmative response to these specific conditions was interpreted as an indication of the presence of CVD.

### 2.3 Covariates

In this study, a comprehensive array of covariates was analyzed, including age, gender, racial background, educational attainment, the ratio of family income to poverty (PIR), body mass index (BMI), estimated Glomerular Filtration Rate (eGFR), and consumption patterns of alcohol and tobacco. Additionally, clinical conditions such as hyperlipidemia, hypertension, and diabetes mellitus were considered. Educational levels were categorized into three groups: less than high school, high school or equivalent, and college education or higher. PIR was stratified into low (PIR < 1.3), middle (≥1.3 and <3.5), and high-income (PIR ≥ 3.5) brackets (23). Alcohol consumption was defined as excessive at four or more drinks per day for men and three or more for women, moderate at two drinks per day for women and three for men, with any additional intake considered minimal (24). Smoking status was determined by a history of consuming over a hundred cigarettes in a year (25). Hyperlipidemia was defined using specific thresholds: total cholesterol ≥200 mg/dl, triglycerides ≥150 mg/dl, high-density lipoprotein ≤40 mg/dl in men and ≤50 mg/dl in women, or low-density lipoprotein ≥130 mg/dl (26, 27). Hypertension was identified by the use of antihypertensive medications, a systolic blood pressure of 140 mmHg or higher, diastolic blood pressure of 90 mmHg or higher, or a documented history of hypertension. Diabetes diagnosis was based on a fasting plasma glucose level of 126 mg/dl or higher, glycated hemoglobin (HbA1c) values of 6.5% or higher, or the use of insulin or hypoglycemic medications. All measured clinical parameters and laboratory data were meticulously collected for the research.

### 2.4 Statistical analysis

We conducted a comprehensive weighting of the study data, adhering to the NHANES guidelines for complex sampling weight calculations, to ensure robust and representative statistical analysis. Continuous variables were presented as means [95% confidence intervals (CI)], and categorical variables were presented as proportions (95% CI). The method of multiple imputation was utilized for the variables that lacked data. Individuals were stratified into quartiles based on their weighted CDAI for baseline characteristic analysis. Using weighted generalized linear models to analyze the linear association between CVD and CDAI. Model 1 was unadjusted; Model 2 accounted for sex, age, and race; Model 3 further controlled for education, PIR, smoking status, alcohol consumption, hypertension, hyperlipidemia, and diabetes. Additionally, we employed restricted cubic spline curves to assess the nonlinear association between the CDAI and CVD. We stratified the analysis by gender and performed threshold effect analysis to identify critical inflection points in this association. Weighted subgroup analyses were conducted to assess the stability of the association between CDAI and cardiovascular disease. Ultimately, we corroborated our findings through an unweighted logistic regression analysis. The R studio (Version 4.2.2) and Empower Stats (version 4.1) were used for all statistical analyses. A two-sided *P* < 0.05 was used to establish statistical significance.

## 3 Results

### 3.1 Initial characteristics of individuals

This study encompassed 25,997 participants, characterized by an average age of 49.72 ± 17.69 years, with a slight majority of females (51.65%) over males (48.35%). The median CDAI is −0.66, with a cardiovascular disease prevalence of 11.01%. As shown in Table 1, the participants were divided into quartiles based on CADI values. A notable trend emerged, showing a decreasing prevalence of cardiovascular disease with increasing CDAI quartiles, a difference that proved to be statistically significant (Quartile 1: 11.57%, Quartile 2: 8.76%, Quartile 3: 8.96%, Quartile 4: 6.79%, *P* < 0.001). Furthermore, statistically significant variations were observed among the four quartiles in terms of age, gender, race, education level, PIR, BMI, smoking habits, alcohol consumption level, eGFR, hyperlipidemia, hypertension, and diabetes mellitus (*P* < 0.05).

**Table 1:** Initial characteristics of individuals according to CADI quartiles.

	Q 1	Q 2	Q 3	Q 4	*P*-value
	*N* = 6,491	*N* = 6,491	*N* = 6,502	*N* = 6,513	
Age(years)	48.55 (47.79, 49.32)	48.52 (47.82, 49.23)	48.11 (47.27, 48.96)	46.12 (45.31, 46.93)	<0.001
Male (%)	33.44 (31.83, 35.09)	42.05 (40.42, 43.70)	50.09 (48.33, 51.86)	64.08 (62.45, 65.68)	<0.001
Race (%)					<0.001
Mexican American	7.12 (5.59, 9.02)	7.98 (6.59, 9.63)	9.12 (7.52, 11.03)	9.41 (7.62, 11.56)	
Other Hispanic	7.19 (5.90, 8.75)	6.55 (5.48, 7.82)	6.57 (5.43, 7.94)	6.53 (5.51, 7.73)	
Non-Hispanic White	62.17 (58.24, 65.95)	64.57 (61.11, 67.89)	66.20 (62.65, 69.58)	64.32 (60.88, 67.61)	
Non-Hispanic Black	14.84 (12.52, 17.50)	12.11 (10.16, 14.39)	9.18 (7.72, 10.89)	10.24 (8.72, 11.98)	
Other race	8.68 (7.55, 9.96)	8.78 (7.67, 10.02)	8.92 (7.74, 10.25)	9.51 (8.27, 10.90)	
Education (%)					<0.001
Less than high school	18.00 (16.34, 19.79)	14.21 (12.77, 15.79)	12.59 (11.25, 14.06)	11.12 (9.68, 12.74)	
High school or equivalent	28.45 (26.62, 30.35)	23.52 (21.73, 25.40)	20.74 (19.00, 22.60)	20.22 (18.66, 21.88)	
College or above	53.55 (51.05, 56.03)	62.27 (59.83, 64.64)	66.67 (64.17, 69.08)	68.66 (66.17, 71.04)	
PIR (%)					<0.001
<1.3	26.71 (24.85, 28.64)	19.62 (17.82, 21.54)	17.38 (15.80, 19.08)	17.51 (15.85, 19.29)	
1.3–3.4	42.16 (40.24, 44.10)	43.78 (41.74, 45.83)	39.05 (37.04, 41.10)	37.76 (35.39, 40.19)	
≥3.5	31.14 (28.80, 33.57)	36.61 (34.32, 38.96)	43.57 (40.94, 46.23)	44.73 (41.57, 47.94)	
BMI (kg/m^2^)	29.58 (29.33, 29.83)	29.73 (29.44, 30.01)	29.29 (29.00, 29.59)	28.84 (28.53, 29.15)	<0.001
Smoking (%)	46.69 (44.99, 48.39)	42.23 (40.33, 44.15)	42.25 (40.19, 44.33)	42.27 (40.40, 44.15)	<0.001
Drinking (%)					<0.001
Light drinking	36.73 (35.22, 38.27)	47.63 (45.99, 49.27)	49.35 (47.54, 51.16)	53.03 (51.12, 54.95)	
Moderate drinking	43.73 (42.15, 45.32)	35.38 (33.90, 36.89)	33.07 (31.34, 34.84)	27.11 (25.69, 28.57)	
Heavy drinking	19.54 (18.23, 20.93)	16.99 (15.55, 18.54)	17.59 (16.06, 19.22)	19.86 (18.00, 21.86)	
eGFR (ml/min/1.73 m^2^)	93.04 (92.06, 94.3)	94.09 (93.07, 95.11)	94.85 (93.85, 95.85)	96.07 (95.18, 96.96)	<0.001
Hyperlipidemia (%)	61.28 (59.39, 63.14)	60.19 (58.35, 62.01)	56.21 (53.61, 58.78)	55.93 (53.93, 57.91)	<0.001
Hypertension (%)	42.33 (40.42, 44.25)	41.47 (39.55, 43.43)	37.80 (36.13, 39.50)	34.79 (32.63, 37.02)	<0.001
Diabetes (%)	16.43 (15.01, 17.95)	14.81 (13.45, 16.28)	13.86 (12.90, 14.88)	12.25 (11.15, 13.44)	<0.001
Cardiovascular disease (%)	11.57 (10.39, 12.86)	8.76 (7.84, 9.77)	8.96 (7.94, 10.09)	6.79 (5.96, 7.72)	<0.001

Means (95% CI) were used to represent continuous variables, while proportions (95% CI) were used to represent categorical data.

### 3.2 Association of the composite dietary antioxidant index with cardiovascular disease

**Figure 2:**
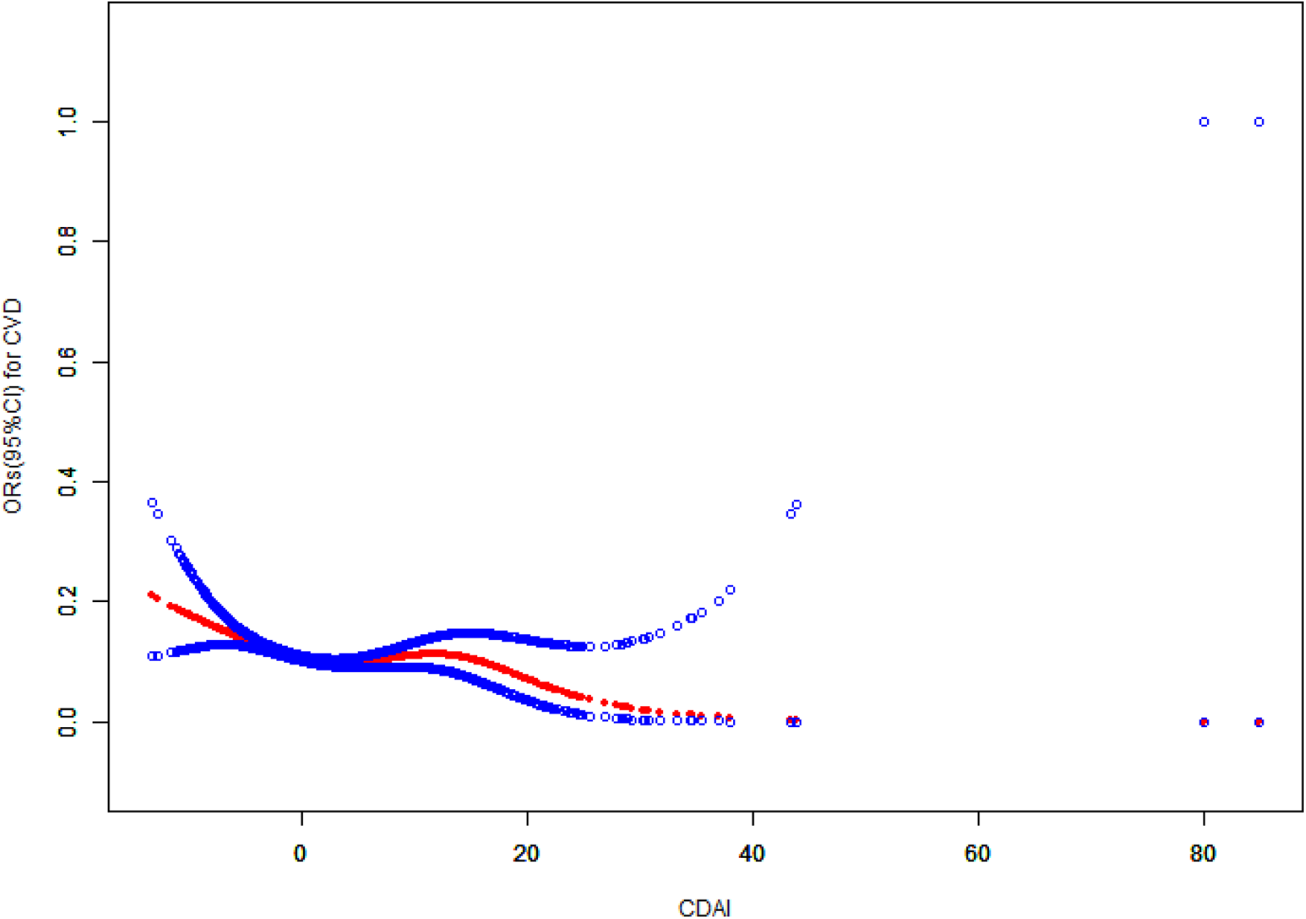
The association between CDAI and cardiovascular disease. CDAI, composite dietary antioxidant index; CVD, cardiovascular disease; CI, confidence interval; OR, odds ratio.

**Figure 3:**
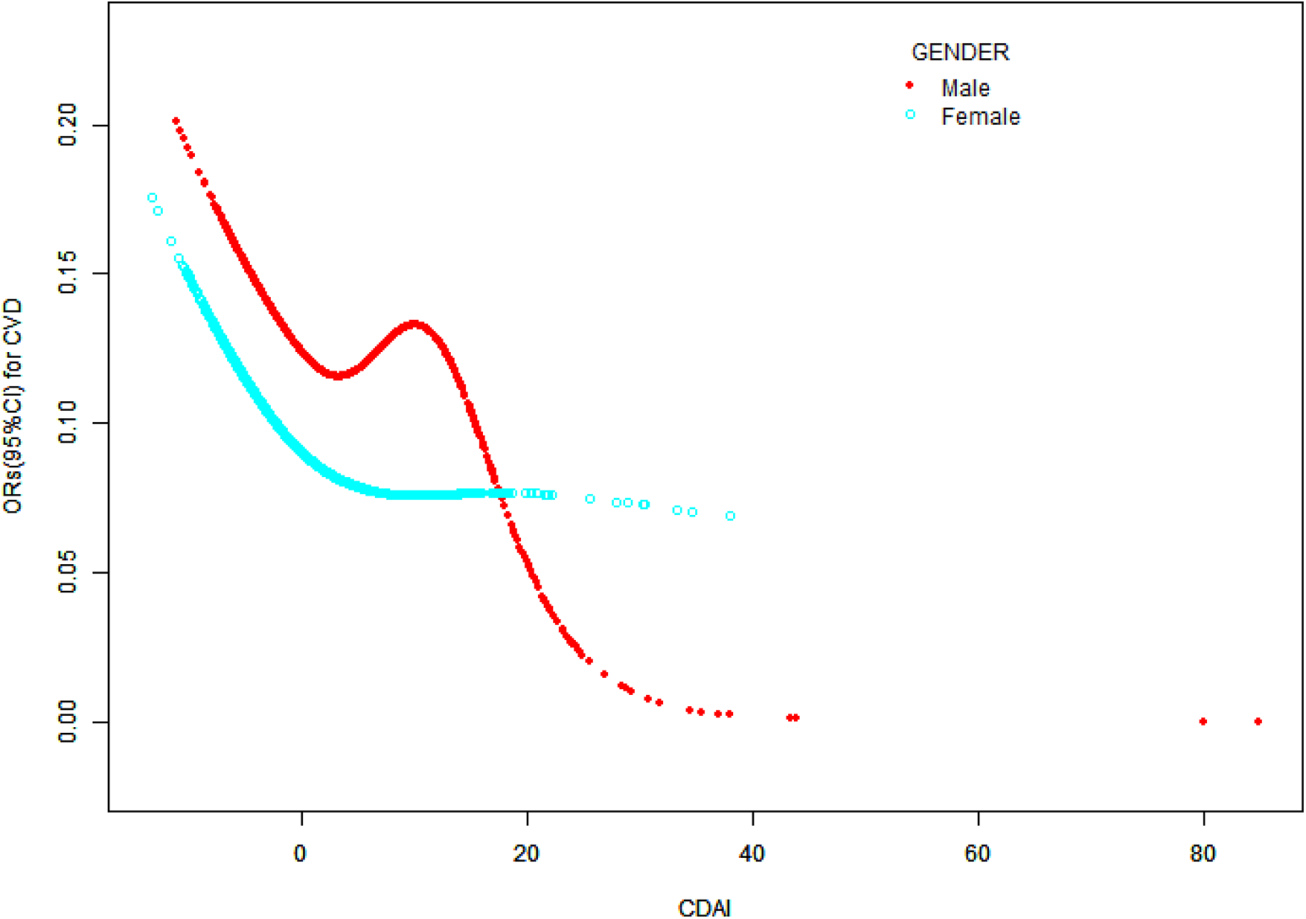
The association between CDAI and cardiovascular disease stratified by sex CDAI, composite dietary antioxidant index; CVD, cardiovascular disease; CI, confidence interval; OR, odds ratio.

The generalized linear regression weighted model of CDAI and CVD is displayed in Table 2. This negative correlation association is evident when CDAI is treated as a continuous variable, manifesting in unadjusted, partially adjusted models with statistical significance (*P* < 0.001). Specifically, in Model 1, Model 2, and Model 3, the fourth quartile exhibited a lower prevalence of cardiovascular disease compared to the first quartile, when CDAI was segmented into quartiles (*P* < 0.001). Notably, in Model 3, the prevalence in Quartile 4 was 29% lower than in Quartile 1 [0.71 (0.59, 0.85), *P* < 0.001], and the trend test was also significant (*P* for trend <0.05). Furthermore, Figure 2 illustrates a nonlinear association between CDAI and cardiovascular disease. Figure 3 shows the base on gender nonlinear association between cardiovascular disease and CDAI. Additionally, Table 3 demonstrates a statistically significant negative association between the prevalence of CVD and the CDAI, particularly in both women and the overall population (*P* < 0.05). Specifically, in women, when the CDAI is less than 3.05, and for every unit increase in CDAI, the prevalence of CVD decreased by 6% (OR = 0.94, 95% CI: 0.90–0.98, *P* = 0.005).

**Table 2:** The association between weighted CDAI and cardiovascular disease.

	Model 1	Model 2	Model 3
	OR (95 CI%) *P*-value	OR (95 CI%) *P*-value	OR (95 CI%) *P*-value
CDAI continuous	0.95 (0.93, 0.97) <0.001	0.96 (0.94, 0.98) <0.001	0.98 (0.96, 1.00) 0.052
CDAI quartiles			
Quartile 1	1	1	1
Quartile 2	0.73 (0.63, 0.85) <0.001	0.69 (0.59, 0.81) <0.001	0.76 (0.64, 0.89) 0.001
Quartile 3	0.75 (0.63, 0.90) 0.003	0.71 (0.59, 0.86) <0.001	0.86 (0.71, 1.04) 0.127
Quartile 4	0.56 (0.48, 0.65) <0.001	0.57 (0.48, 0.67) <0.001	0.71 (0.59, 0.85) <0.001
*P* for trend	<0.001	<0.001	0.007

Model 1 was unadjusted for variables, model 2 was adjusted for sex, age, and race, and model 3 was adjusted for the above factors smoking, alcohol consumption, education, PIR, BMI, hyperlipidemia, diabetes, and hypertension.

**Table 3:** Weighted analysis of threshold effects of CDAI on cardiovascular disease.

CDAI	Adjusted OR 95% CI, *P*-value
Inflection point	
CDAI <0.36	0.94 (0.89, 0.98) 0.010
CDAI >0.36	1.00 (0.97, 1.04) 0.834
Log likelihood ratio	0.002
Male	
CDAI ≤0.23	0.96 (0.87, 1.05) 0.371
CDAI ≥0.23	1.00 (0.97, 1.03) 0.912
Log likelihood ratio	0.033
Female	
CDAI <3.05	0.94 (0.90, 0.98) 0.005
CDAI >3.05	1.05 (0.98, 1.12) 0.142
Log likelihood ratio	0.041

### 3.3 Subgroup investigation of the connection between the composite dietary antioxidant index and cardiovascular disease

According to Table 4, in our subgroup analysis, which considered variables such as gender, age, race, education level, PIR, BMI, and habits like smoking and drinking, along with hyperlipidemia, diabetes, and hypertension, the negative association between CDAI and cardiovascular disease persisted across all categories. Nevertheless, there was a significant interaction between the CDAI and CVD in the smoking subgroup analysis (*P* for interaction <0.05).

**Table 4:** Subgroup analysis of the association between weighted CDAI and cardiovascular disease.

	Q1	Q2	Q3	Q4	*P* for interaction
Gender					0.107
Male	Ref	0.87 (0.69, 1.10) 0.245	0.95 (0.74, 1.21) 0.667	0.85 (0.66, 1.10) 0.226	
Female	Ref	0.69 (0.56, 0.86) 0.002	0.82 (0.65, 1.04) 0.106	0.55 (0.41, 0.73) <0.001	
Age					0.221
<50	Ref	0.62 (0.42, 0.93) 0.024	0.57 (0.38, 0.86) 0.010	0.50 (0.30, 0.86) 0.015	
≥50	Ref	0.76 (0.64, 0.90) 0.002	0.90 (0.73, 1.11) 0.320	0.73 (0.61, 0.87) <0.001	
Race					0.103
Mexican American	Ref	0.77 (0.45, 1.30) 0.332	0.78 (0.54, 1.12) 0.185	0.54 (0.32, 0.89) 0.021	
Other Hispanic	Ref	0.82 (0.53, 1.27) 0.390	0.99 (0.56, 1.75) 0.977	0.57 (0.35, 0.92) 0.028	
Non-Hispanic White	Ref	0.67 (0.54, 0.83) <0.001	0.82 (0.65, 1.04) 0.107	0.67 (0.53, 0.85) 0.002	
Non-Hispanic Black	Ref	1.02 (0.82, 1.27) 0.881	0.88 (0.67, 1.17) 0.390	0.91 (0.69, 1.19) 0.495	
Other Race	Ref	1.21 (0.64, 2.31) 0.560	1.09 (0.62, 1.89) 0.774	0.96 (0.55, 1.69) 0.892	
Education level					0.383
Less than high school	Ref	0.81 (0.63, 1.04) 0.111	0.68 (0.50, 0.93) 0.018	0.70 (0.49, 1.02) 0.071	
High school or equivalent	Ref	0.72 (0.55, 0.95) 0.026	0.84 (0.57, 1.24) 0.390	0.61 (0.41, 0.91) 0.020	
College or above	Ref	0.76 (0.60, 0.96) 0.029	0.94 (0.75, 1.18) 0.586	0.77 (0.60, 0.99) 0.044	
PIR					0.370
<1.3	Ref	0.81 (0.58, 1.11) 0.198	0.68 (0.51, 0.92) 0.017	0.64 (0.46, 0.89) 0.011	
≥1.3 and <3.5	Ref	0.73 (0.58, 0.91) 0.008	0.95 (0.74, 1.24) 0.727	0.72 (0.56, 0.92) 0.012	
≥3.5	Ref	0.77 (0.49, 1.22) 0.275	0.85 (0.57, 1.28) 0.450	0.75 (0.55, 1.01) 0.066	
BMI					0.731
<25	Ref	0.82 (0.59, 1.15) 0.261	0.90 (0.60, 1.37) 0.633	0.76 (0.52, 1.11) 0.160	
≥25and <30	Ref	0.75 (0.57, 1.00) 0.058	0.90 (0.65, 1.24) 0.519	0.84 (0.62, 1.14) 0.279	
≥30	Ref	0.73 (0.58, 0.92) 0.010	0.81 (0.61, 1.08) 0.158	0.60 (0.45, 0.78) <0.001	
Smoking					0.003
Yes	Ref	0.72 (0.58, 0.89) 0.004	0.68 (0.53, 0.86) 0.002	0.70 (0.56, 0.87) 0.002	
No	Ref	0.81 (0.63, 1.03) 0.096	1.16 (0.90, 1.50) 0.269	0.72 (0.53, 0.97) 0.034	
Drinking					0.977
Light drinking	Ref	0.71 (0.57, 0.90) 0.007	0.88 (0.68, 1.13) 0.325	0.71 (0.57, 0.88) 0.004	
Moderate drinking	Ref	0.80 (0.58, 1.11) 0.185	0.85 (0.65, 1.11) 0.231	0.68 (0.50, 0.92) 0.016	
Heavy drinking	Ref	0.81 (0.51, 1.28) 0.365	0.75 (0.44, 1.27) 0.292	0.73 (0.44, 1.22) 0.242	
Hyperlipidemia					0.171
Yes	Ref	0.70 (0.57, 0.86) 0.002	0.73 (0.58, 0.93) 0.014	0.66 (0.53, 0.82) <0.001	
No	Ref	0.85 (0.69, 1.05) 0.146	1.07 (0.81, 1.41) 0.630	0.80 (0.61, 1.04) 0.102	
Hypertension					0.720
Yes	Ref	0.75 (0.61, 0.91) 0.006	0.80 (0.63, 1.02) 0.083	0.71 (0.57, 0.88) 0.003	
No	Ref	0.78 (0.58, 1.04) 0.102	1.01 (0.71, 1.42) 0.973	0.71 (0.49, 1.03) 0.074	
Diabetes					0.409
Yes	Ref	0.70 (0.53, 0.93) 0.017	0.70 (0.53, 0.93) 0.018	0.67 (0.50, 0.90) 0.010	
No	Ref	0.79 (0.64, 0.97) 0.028	0.94 (0.74, 1.20) 0.637	0.73 (0.58, 0.92) 0.009	

### 3.4 Sensitivity analysis

Sensitivity analyses conducted with unweighted logistic regression, as presented in Table 5, reveal a consistent negative association between CDAI and CVD prevalence across Model 1, Model 2, and Model 3. These findings align with those obtained from the weighted logistic regression analysis.

**Table 5:** The association between CDAI and cardiovascular disease in sensitivity analysis using unweighted logistic regression analysis.

	Model 1	Model 2	Model 3
	OR (95 CI%) *P*-value	OR (95 CI%) *P*-value	OR (95 CI%) *P*-value
CDAI Continuous	0.95 (0.94, 0.96) <0.001	0.96 (0.94, 0.97) <0.001	0.97 (0.96, 0.98) <0.001
CDAI quartiles			
Quartile 1	1	1	1
Quartile 2	0.79 (0.71, 0.87) <0.001	0.78 (0.69, 0.87) <0.001	0.83 (0.74, 0.93) 0.002
Quartile 3	0.69 (0.62, 0.77) <0.001	0.70 (0.62, 0.78) <0.001	0.79 (0.70, 0.89) <0.001
Quartile 4	0.57 (0.51, 0.64) <0.001	0.62 (0.55, 0.70) <0.001	0.73 (0.64, 0.83) <0.001
*P* for trend	<0.001	<0.001	<0.001

Model 1 was unadjusted for variables, model 2 was adjusted for sex, age, and race, and model 3 was adjusted for the above factors smoking, alcohol consumption, education, PIR, BMI, hyperlipidemia, diabetes, and hypertension.

## 4 Discussion

To the best of our knowledge, our investigation represents the inaugural study exploring the association between CDAI values and the prevalence of cardiovascular disease. This study encompassed a cohort of 25,997 individuals from the 2011 to 2020 NHANES database. Our findings reveal a nonlinear association between CDAI and CVD prevalence. This negative association appeared robust and unaffected by variables such as gender, age, race, education level, PIR, BMI, alcohol consumption, hyperlipidemia, hypertension, and diabetes (*P* > 0.05). This consistency underlines the reliability and stability of the negative association between CDAI and CVD prevalence. Furthermore, our analysis of women identified an inflection point in the CDAI and cardiovascular disease association at a CDAI value of 3.05. Intriguingly, below this threshold, each unit increase in CDAI corresponded to a 6.0% decrease in CVD prevalence (*P* < 0.05).

OS has been recognized as one of the risk factors for cardiovascular disease. However, single antioxidant nutrients do not exhibit uniform associations with cardiovascular disease. A meta-analysis indicated that vitamin C, vitamin E, and β-carotene may potentially reduce the risk of cardiovascular disease mortality (28). Another meta-analysis of 884 randomized controlled intervention trials showed that individual vitamins C, D, E, and selenium did not impact cardiovascular disease incidence significantly. In contrast, β-carotene supplementation increased all-cause mortality, CVD mortality, and stroke risk (29). A study by Nazari suggests that zinc supplementation may increase the risk of coronary atherosclerosis (30), but further investigation is needed to understand the exact mechanism. A randomized controlled trial confirms that vitamin A and vitamin D supplementation improves clinical prognosis in patients with ischemic stroke (31). It is important to note that maintaining normal cellular function depends on the balance between OS and antioxidant capacity, and excessive intake of antioxidant nutrients may have adverse effects (32). The amount of antioxidants needed for protection may vary among individuals, influenced by factors such as gender, age, and lifestyle habits (33). Our study demonstrates that the U-shaped association between CDAI and cardiovascular disease exists only in women, consistent with the findings of Liu, who reported a similar L-shaped association between CDAI levels and atherosclerotic cardiovascular disease in postmenopausal women (34).

There was a significant interaction between CDAI quartiles and CVD prevalence in the smoking subgroup analysis. (*P* for interaction = 0.003). This may be because smoking exacerbates cardiovascular disease risk via mechanisms such as heightened oxidative stress, inflammatory responses, and impaired endothelial function (35). In concert with CDAI, these factors intensify the association in smokers. Higher CDAI levels appeared to have a more significant impact on cardiovascular health in smoking populations compared to non-smoking populations. Smoking may serve as an amplifying factor for the effects of CDAI on cardiovascular health. However, further prospective research is needed to determine the exact mechanism underlying this behavior. Meanwhile, the pathogenesis of cardiovascular disease due to oxidative stress is complex, and further research is warranted to confirm these conclusions. Different antioxidants may exhibit synergistic or antagonistic effects on cardiovascular disease, influencing its development through its ability to combat OS (36). In contrast, CDAI is an indicator that combines six antioxidant nutrients, providing a comprehensive assessment of their impact on cardiovascular health (37, 38).

Our study has several strengths: firstly, it is the first to investigate the association between CDAI and cardiovascular disease in the general population; secondly, our model adjusted for potential confounders as comprehensively as possible; and thirdly, our study utilized the NHANES database, which provided health- and nutrition-related data on 25,997 participants, enhancing the reliability of our results. Nevertheless, our study has certain limitations. First of all, Variations across different periods might influence the CDAI, variables, and the prevalence of cardiovascular disease. Although we adhered to NHANES guidelines for calculating complex sample weights, we cannot entirely dismiss the potential impact on our findings. And because the study is cross-sectional, it is more challenging to determine the causative links between CDAI and CVD risks. Additional prospective research is required to validate our results. Secondly, we may not have accounted for all potential confounders despite adjusting for multiple covariates. Thirdly, the results mainly apply to the American population because racial dietary patterns range significantly, and further multiethnic research is required to investigate the association between CDAI and cardiovascular disease.

## 5 Conclusion

In conclusion, our study confirms the negative association between CDAI and CVD prevalence in the general population, and women, the inflection point was 3.05. However, we need more prospective studies to explore further.

## Data Availability

Publicly available datasets were analyzed in this study. This data can be found: https://wwwn.cdc.gov/nchs/nhanes/.
